# Translational Approach to the Protective Effect of Bilirubin in Diabetic Kidney Disease

**DOI:** 10.3390/biomedicines10030696

**Published:** 2022-03-17

**Authors:** Annalisa Bianco, Claudio Tiribelli, Cristina Bellarosa

**Affiliations:** 1 Italian Liver Foundation (FIF), 34149 Trieste, Italy; annalisa.bianco@ba.itb.cnr.it (A.B.); ctliver@fegato.it (C.T.); 2 National Research Council, Institute of Biomedical Technologies, Bari Unit, 70126 Bari, Italy

**Keywords:** bilirubin, diabetic kidney disease, oxidative stress, inflammation, fibrosis, apoptosis

## Abstract

Bilirubin has been regarded as a powerful endogenous antioxidant and anti-inflammatory molecule, able to act on cellular pathways as a hormone. Diabetic kidney disease (DKD) is a common chronic complication of diabetes, and it is the leading cause of end-stage renal disease. Here, we will review the clinical and molecular features of mild hyperbilirubinemia in DKD. The pathogenesis of DKD involves oxidative stress, inflammation, fibrosis, and apoptosis. Serum bilirubin levels are positively correlated with the levels of the antioxidative enzymes as superoxide dismutase, catalase, and glutathione peroxidase, while it is inversely correlated with C-reactive protein, TNF-α, interleukin (IL)-2, IL-6, and IL-10 release in diabetic kidney disease. Bilirubin downregulates NADPH oxidase, reduces the induction of pro-fibrotic factor HIF-1α expression, cleaved caspase-3, and cleaved PARP induction showing lower DNA fragmentation. Recent experimental and clinical studies have demonstrated its effects in the development and progression of renal diseases, pointing out that only very mild elevations of bilirubin concentrations result in real clinical benefits. Future controlled studies are needed to explore the precise role of bilirubin in the pathogenesis of DKD and to understand if the use of serum bilirubin levels as a marker of progression or therapeutic target in DKD is feasible and realistic.

## 1. Introduction

Historically bilirubin was considered only as a non-functional, waste product of heme catabolism, a sign of liver disease or, even worst, potentially neurotoxic molecules. Since mammals spend energy and resources converting biliverdin (a non-toxic compound that is relatively easy to secrete) into bilirubin (which needs to be further metabolized for its excretion via a biliary system), it is reasonable to believe that bilirubin is more than just a degradation product of heme catabolism. In the last decades, clinicians began to notice mild hyperbilirubinemia presented by Gilbert Subjects (but also levels in the upper quartiles of the currently accepted physiological serum bilirubin range) protected against the increase of civilization diseases (cardiovascular diseases, diabetes, obesity, metabolic syndrome) based mainly on oxidative stress. Meanwhile, basic scientists began to investigate the mechanisms involved in the protective role of this molecule, and together with the bilirubin antioxidant capacity as an ROS scavenger, they discovered that bilirubin is an important modulator of various biological functions in the human body, and it is able to act as a hormone directly targeting its receptor to exert its effect [1]. Since a tiny increase of serum bilirubin levels seems to significantly reduce the impact of oxidative stress-based diseases, scientists are taking into consideration the possibility to increase serum bilirubin levels as a preventive method against civilization diseases [2].

Diabetic kidney disease (DKD) occurs in approximately 20% to 40% of patients with type 1 or type 2 diabetes mellitus. Early diagnosis of DKD is fundamental to avoid the progression to end-stage renal failure. Current research is focused on finding methods to predict DKD progression and improving the treatment [3].

Endogenous bilirubin appeared crucial both as a potential marker for progression and as a therapeutic target for the prevention of DKD. According to different recent studies well explained in the present review, total serum bilirubin level could be considered a marker of DKD progression, helpful in detecting low- and high-risk patient groups. Patients with a low–normal total bilirubin concentration may be managed aggressively to delay the progression to kidney failure. Bilirubin as a marker has the advantage to be measured easily, inexpensively, and routinely in most medical centers. Further studies are required to determine whether the total bilirubin concentration is a potential therapeutic target for the prevention of CKD. There is a plethora of measures able to mildly increase serum bilirubin levels, including lifestyle changes, the use of natural compounds as nutraceuticals or chemical drugs, and the possibility to include bilirubin in nanoparticles [4].

In the present work, we will review the clinical effects of mild hyperbilirubinemia against civilization diseases (with a focused attention on DKD) and the related molecular mechanisms and pathways involved. This evidence is necessary to better understand if the use of serum bilirubin levels as a marker of progression or therapeutic target in DKD is feasible and realistic.

## 2. Bilirubin

### 2.1. Bilirubin Metabolism

Bilirubin belongs to the tetrapyrrolic compounds superfamily, and it is the end product of heme catabolism occurring mainly in the splenic reticuloendothelial system [5]. A healthy adult produces around 4.4 ± 0.7 mg/Kg by bodyweight of bilirubin daily [6]. The senescent erythrocytes are the principal sources of the heme group but not the only ones; although approximately 80% of this quantity derives from hemoglobin of senescent red cells, the remaining part is shared between the turnover of myoglobin (another protein containing the heme group and specialized into O_2_ transport), cytochromes, and other hemoproteins, such as microsomal cytochrome CYP-450 [7]. Altogether, these proteins provide the 15–20% of the available substrate; lastly, the destruction of immature red blood cells in the bone marrow donates 3% of the heme group [8].

**Figure 1:**
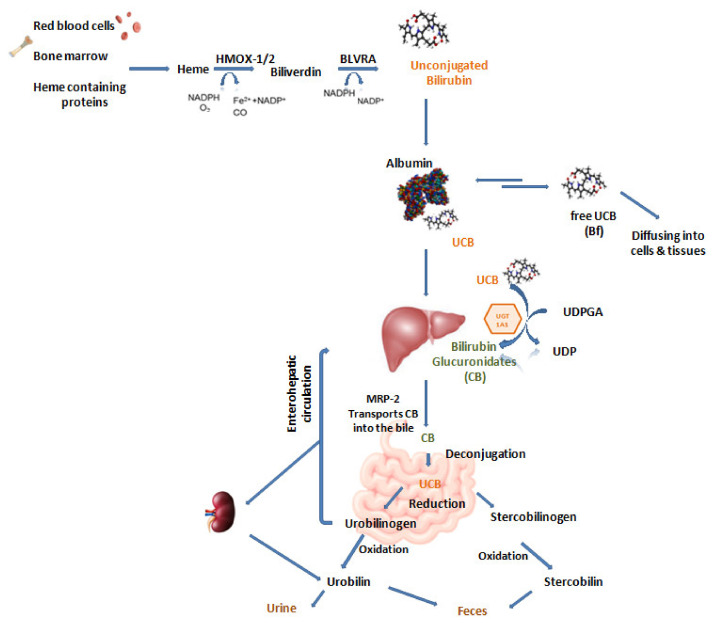
Bilirubin metabolism. HMOX-1/2: hemeoxygenase enzyme; NADPH: nicotinamide adenine dinucleotide phosphate reduced form; O_2_: oxygen; Fe^2+^: ferrous ions; NADP^+^: nicotinamide adenine dinucleotide phosphate oxidized form; CO: carbon monoxide; BLVRA: biliverdin reductase A enzyme; UCB: unconjugated bilirubin; Bf: free bilirubin; UGT1A1: uridine diphosphate-glucuronosyl transferase 1A1; UDPGA: uridine 5′-diphosphoglucuronic acid; UDP: uridine diphosphate; CB: conjugated bilirubin;MRP-2:multidrug resistance-associated protein 2.

Bilirubin metabolism consists of several steps, including production, uptake by the hepatocyte, conjugation, excretion into bile ducts, and delivery to the intestine (Figure 1). Jaundice can result from defects in any of these steps of bilirubin metabolism.

First, the heme group is converted by the heme oxygenase 1 enzyme (HMOX, Online Mendelian Inheritance in Man (OMIM) 1 No.*141250) into biliverdin releasing CO, Fe^2+^, H_2_O, and contemporary oxidating NADPH into NADP. This enzyme induces the opening of the heme ring, the freeing of iron ion, and the formation of a tetrapyrrolic chain. Biliverdin is subsequently reduced to bilirubin by cytosolic enzyme biliverdin reductase (BLVRA, OMIM No.*109750) in the presence of NADPH [5].

Since bilirubin is highly insoluble in water (the solubility threshold in plasma is as low as 70 nmol/L = 0.004 mg/dL) [9], it can be carried in the vascular bed by the binding to albumin, the most abundant protein in the blood. Due to the strong binding affinity (Ka = 7 × 10^7^/M) and the high concentration of albumin in human serum, most bilirubin is bound to albumin, and just less than 0.1% of bilirubin remains unbound and, therefore, named free bilirubin (Bf). The pathophysiological properties of this pigment are related to the fraction of free bilirubin rather than total bilirubin [10,11]. Thanks to the albumin binding, bilirubin can reach the liver where the pigment is actively transported inside the hepatocytes at the sinusoidal membrane via the OATP1B1, organic anion transporting polypeptide 1B1 (OMIM No.*604843) [6]. Once in hepatocytes cytoplasm, bilirubin is solubilized by specific binding proteins of which the two most interesting are protein Y (ligandin, α isoform of glutathione S-transferase B) and protein Z (liver-specific fatty acid-binding protein, FABP 1) and directed to the endoplasmic reticulum [8].

Then, bilirubin in the liver is conjugated with one or two molecules of glucuronic acid by the UDP-glucuronosyltransferase 1A1 (UGT1A1) enzyme [12]. Conjugated bilirubin (CB) is efficiently secreted into bile, mostly by the ATP-dependent MRP2/ABCC2 transporter (OMIM No.*601107) and spilled in the duodenum [13]. Within the intestine, almost all bilirubin is deconjugated. The majority is excreted as urobilinoids [14], while the other part is excreted as unconjugated bilirubin (UCB). Firstly, CB is deconjugated into UCB by beta-glucuronidase, and then microbiota reduces UCB to urobilinogen and stercobilinogen. Stercobilin and urobilin are excreted via feces. Physiologically, a small fraction of UCB and urobilinogenis reabsorbed by enterohepatic circulation. Urobilinogen moves to the kidney to be filtered, and then it is excreted through the urine [7]. UCB is reabsorbed in the colon and returned to the liver by the portal circulation [15].

Defects in UCB hepatic uptake and conjugation increase serum UCB levels, with a consequent rise of the blood Bf fraction content [16].

### 2.2. Bilirubin as Janus Bifrons

Bilirubin behavior in a human body has two faces, similar to the Janus Bifrons, a Roman god. Severe hyperbilirubinemia lasting for a long time leads to neuronal injury or bilirubin encephalopathy (kernicterus). Kernicterus is usually characterized by choreoathetoid cerebral palsy, impaired upward gaze, and sensorineural hearing loss, whereas cognition is relatively spared. Prolonged and severe unconjugated hyperbilirubinemia is responsible for kernicterus due to the ability of the Bf to cross the blood-brain barrier and to precipitate into the brain as it is neurotoxic [17,18]. Unconjugated hyperbilirubinemia could be caused by an increase of UCB production or by a less effective hepatic uptake or conjugation of bilirubin. The most common conditions of unconjugated hyperbilirubinemia are Crigler-Najjar syndromes (CNS) type 1 and 2. CNS type 1 patients frequently present kernicterus and a complete loss of UGT1A1 activity, while in CNS type 2 patients, the enzyme works partially due to a missense mutation [19]. The toxic effects of bilirubin are exploited by inhibition of DNA synthesis, RNA synthesis, protein synthesis in the brain and liver, and the alteration of carbohydrate metabolism in the brain [11,16,17]. A model for inherited deficiency of bilirubin glucuronidation is the Gunn rat. It is a natural mutant rat discovered by Dr. Gunn in 1934. These rats are jaundiced and present unconjugated hyperbilirubinemia due to natural mutation that causes the lack of the enzyme uridine diphosphate glucuronosyltransferase. It was transmitted as an autosomal recessive characteristic. The Gunn rat is a natural model for bilirubin encephalopathy [20,21], and much of the knowledge of bilirubin toxicity and its treatment has come from studies performed in these rats [16,22]. However, the Gunn rat represents a well-described animal model to investigate not only the neuronal-damaging effects of hyperbilirubinemia in neonates [16,22], but also the systemic protective effects of mild hyperbilirubinemia in adults [16,23,24,25,26].

Although its excessive accumulation can cause permanent brain damage, more recent studies recognized this bile pigment has numerous beneficial effects when its level is mildly elevated [5,27]. These effects have been shown in patients with Gilbert’s Syndrome (GS). GS is a benign condition caused by a partial deficiency of hepatic bilirubin UDP glucuronosyltransferase (UGT1A1) without any signs of liver damage [16,28]. The addition of an extra dinucleotide sequence, TA, to the subsequent TATA box promoter of UGT1A1 (known as the UGT1A1 * 28 allele) would appear to be the most common molecular defect in GS. Homozygous subjects show ~10–35% UGT1A1 activity compared to normal subjects. Clinically they present random hyperbilirubinemia, usually associated with prolonged fasting, fever, and important physical exercise. GS is the most common hereditary jaundice, and the prevalence of GS in the population is ~8%. Since mild hyperbilirubinemia reduces the prevalence of metabolic syndrome, including obesity, overweight, diabetes type II, certain cancers, and cardiovascular diseases (CDV), UGT1A1 mutations may provide a genetic advantage [29]. For these reasons, scientists hypothesized that the high frequency of homozygous genetic mutations of the UGT1A1 gene worldwide could be due in part to evolutionary advantages [30]. Modulation of bilirubin levels may be an attractive issue to work on for metabolic disease treatment and/or prevention. Although Gilbert syndrome is known as a benign condition, the metabolism of some drugs that undergo glucuronidation may be affected in these patients, as UGT1A1 is a hepatic enzyme, e.g., non-steroidal inflammatory drugs, statins, and human immunodeficiency virus protease inhibitors. The reduced glucuronidation capacity of the hepatocytes can result in severe toxicity for the organism [30].

In recent years, the Gunn rat was also utilized to study the protective effects of mild hyperbilirubinemia in adults [16,23,24,25,26]. The hyperbilirubinemic homozygous Gunn rats (jj) maintain serum bilirubin concentrations close to the upper limit of Gilbert subjects, also showing increased bilirubin levels in tissues and organs for their entire lives. UCB concentration in hyperbilirubinemic homozygous Gunn rats (jj) serum (approximately from 2.42 to 7.36 mg/dL) [31,32] overlaps with elevated bilirubin concentrations seen in Gilbert subjects [31,33,34].

In addition, recent research reveals that low plasma bilirubin levels, defined as ‘hypobilirubinemia’, are a possible new pathology analogous to the other end of the spectrum of extreme hyperbilirubinemia. Plasma bilirubin levels lower than 0.6 mg/dL are commonly seen in patients with metabolic dysfunction, which may lead to cardiovascular complications and possibly stroke [35].

### 2.3. Bilirubin Protective Effects

Bilirubin has been regarded as a potent endogenous antioxidant agent. Therefore, in recent years, the role of bilirubin in the possible prevention of oxidative stress-mediated diseases, in particular CVD, was reviewed extensively [2]. Evidence for an in vivo antioxidant capacity of mild hyperbilirubinemia is provided by Gilbert subjects and Gunn rat models. Regarding CVD incidence, Gilbert subjects were shown to have a significantly lower prevalence (2% vs. 12%) compared with the normobilirubinemic population [36,37]. The effects on lipid profile and cholesterol levels contributing to the protective actions of UCB have been demonstrated in individuals with Gilbert’s polymorphism and in animal models of moderate hyperbilirubinemia [38]. Higher serum bilirubin is positively associated with an increased HDL/LDL ratio and protection of these lipids from oxidation, as well as with lowering LDL cholesterol, ApoB/ApoA1, and VLDL. UCB might also protect from metabolic syndrome and diabetes [31,39] and may predict the progression of DKD in patients with type 2 diabetes. Recently, several retrospective observational longitudinal studies performed in Chinese [40,41], Korean [42], and Turkish [43] type 2 diabetes patients showed that total levels of bilirubin are inversely associated with the incidence and progression of diabetic kidney disease (DKD) and suggested that the serum total bilirubin level may be used as a marker of DKD progression. In all these studies, the type 2 diabetic patients with normal total bilirubin levels were divided into three or four groups according to the quartiles of the total serum bilirubin levels at baseline. In all studies, the group with higher bilirubin levels (mg/dL) (G3: 0.6–0.9 [43]; Q3 ≥ 0.82 [41]; Q4: 1–1.13 [40]; Q4 > 0.88 [42]) had the lowest risk of progression of CKD. These findings were confirmed in Chinese patients with type 1 diabetes mellitus [44]. The antioxidant ability of UCB is linked to the redox system converting UCB to biliverdin where the reactive oxidant species (ROS) are consumed and the bilirubin regenerated via BLVR [45]. Conversely, severe hyperbilirubinemia causes ROS production [46], protein oxidation, and lipid peroxidation [47,48], leading to apoptosis [49] in various cellular systems. The antioxidant and pro-oxidant effect of bilirubin are also confirmed by our in vitro comparative study that defined the intracellular bilirubin thresholds that set the switch between bilirubin effects [50].

Anti-inflammatory and immunomodulatory activities by bilirubin were also demonstrated [51]. These effects consist of a cascade of events, including mild hyperbilirubinemia, ER stress, and inflammation [1,52]. Milder elevations of bilirubin concentrations attenuate ER stress and decrease the level of inflammatory cytokines [46,53,54], both in vitro and in vivo [55]. Bilirubin exhibits a significant anti-inflammatory capacity due to mechanisms such as inhibition of adhesion molecule expression, suppression of inflammatory cell infiltration, and reduction of pro-inflammatory cytokine levels in animal models of end otoxemia, septicemia, and injury from ischemia reperfusion. Furthermore, bilirubin has been demonstrated to fight lipopolysaccharides (LPSs), a product of bacteria. LPSs induce liver damage and cardiovascular disease [53,54]. In addition, UCB and HO-1 simultaneously reduce the increasing tumor necrosis factor-α (TNF-α), nitric oxide (NO), iNOS, endothelial dysfunction, and they block proliferation and migration of the cells via Raf/ERK/MAPK pathway and display anti-inflammatory activity as well. Bilirubin affects cell signal transduction to prevent the nuclear translocation of nuclear factor (NF) kappaB induced by TNF-α [27,56]. In an in vitro model of gut inflammation, ERstress and the subsequent inflammatory response were reduced by co-treatment with UCB [57].

Recently, Hinds demonstrated bilirubin hormonal function [58]. Bilirubin directly binds to receptors involved in energy homeostasis implementation (such as peroxisome proliferator-activated receptors [PPARs], aryl-hydrocarbon receptor [AhR], or constitutive and androstane receptor [CAR]), processes of biotransformation (such as CAR, pregnane X receptor (PXR)), or sensitive perception (via MRGPRX4 (Mas-related G protein-coupled receptor X4)) [6,59]. In addition, bilirubin can complex with some molecules involved in energy homeostasis [60], belonging mostly to a lipocalin superfamily of proteins, such as fatty acid-binding protein [FABP1] or apolipoprotein D [apoD] and activate various additional cell-signaling pathways [6,59].

## 3. Diabetic Kidney Disease

DKD (also called “chronic kidney disease” [CKD] due to diabetes or diabetic nephropathy [DN]) [61] is a common chronic complication of diabetes, and it is the majorcause of end-stage renal disease. Up to 40% of type II diabetic patients progress to DKD, and the incidence of this metabolic disorder is increasing rapidly worldwide [62]. In recent years, due to the increase in childhood obesity, the prevalence of type II diabetes is becoming more common in the young population in which DKD manifests itself more aggressively than in the adult population [63]. In addition, increasing evidence has shown that sex and gender differences are implicated in the prevalence of different and specific DKD phenotypes and in the impact and control of common DKD risk factors. Evidence on sex/gender differences in DKD, while considering hormonal, genetic, and clinical factors, keep the door open for a distinct and personalized therapeutic approach [64].

Clinically, proteinuria (excess of serum proteins in the urine) is the main index of diabetic nephropathy; nevertheless, it is not an accurate method to evaluate its severity or prognosis since many patients develop DKD and renal disorders without prior proteinuria. Kidney biopsy, even if it is an invasive procedure, is essential for DKD differential diagnosis and staging of the disease [65]. DKD is also characterized by hypertension, renal failure leading to edema, and uremic symptoms. The functional unit of the kidney is the nephron, consisting of glomerulus, proximal tubule, Henle’s loop, and distal convoluted tubule. A fine circulatory system lets blood to reach the glomeruli, where plasma is filtered into the Bowman’s capsule. The human kidney can filter 180 L of blood through its glomeruli and produce about 2 L of urine daily [66]. DKD affects both tubular and glomerular elements of nephrons leading to glomerulosclerosis, tubulointerstitial fibrosis (TIF), tubular atrophy, podocytes detachment, and apoptosis and results in the loss of normal renal architecture and renal filtration capacity [67]. The pathogenesis of DKD may be multifactorial. The earliest changes are triggered by metabolic factors and are related to uncontrolled or chronic hyperglycemia. However, the roles of various mechanisms have been established, including those of high glucose and advanced glycation end-product (AGE) exposure, the polyol pathway activation, glomerular hyper-filtration, ROS increase, diacylglycerol (DAG)/protein kinase C (PKC) pathway activation, TGF-β signaling, and renin–angiotensin–aldosterone system (RAAS) signaling [68].

Oxidative stress and renal ROS lead to the development of diabetic nephropathy. Physiologically, the kidney generates an important amount of ROS because of its high metabolic activity that is balanced by a wide antioxidant system. ROS are produced by different macromolecules, including NAD(P)H oxidase, AGE, defects in polyol pathway, uncoupled nitric oxide synthase (NOS), and mitochondrial respiratory chain via oxidative phosphorylation. The pathologic states, such as hyperglycemia, shifts the oxidative balance toward a pro-oxidant state that accelerates tissue and vascular damage. Increased ROS modulates the activation of protein kinase C, mitogen-activated protein kinases, and various cytokines and transcription factors, leading to the progression to fibrosis and end-stage renal disease.

Inflammation has a key role in the evolution of kidney disease. During the inflammatory process caused by hyperglycemia, monocytes and lymphocytes penetrate the kidney by secreting proinflammatory cytokines and reactive oxygen species. The inflammatory response is amplified by leukocyte activity that promotes cell damage, leading to fibrosis [69]. Nuclear factor-κB (NF-κB), interleukin-6 (IL-6), TNF-α, transforming growth factor-β (TGF-β), and soluble C-X-C chemokine ligand (CXCLs) are cytokines and chemokines that play essential and crucial roles in the inflammatory response [70]. Basic and clinical studies demonstrate the role of IL-6 signalling in progression of DKD. Serum IL-6 levels increased in diabetic patients with DKD versus those without DKD [71], and experimental evidence indicate that IL-6 damages podocytes in the evolution of DKD, inducing their hypertrophy, and can lead to cell cycle arrest [72].

Renal fibrosis is an end-stage disease change in DKD. Among promoters of kidney fibrosis, recently HIF-1α was identified [73]. In experimental models of chronic kidney diseases, its activation has been demonstrated to stimulate accumulation of collagen and recruitment of inflammatory cells [74,75]. The main stimulus for HIF activation is hypoxia, but HIF-1αis is also activated by Ang II [76]. The pro-fibrotic HIF-1α pathway leading to tubulointerstitial fibrosis maturation involves LOXl2 [77]. HIF-1α regulates biological processes relevant for tissue repair, wound healing, and fibrogenesis. Fibrogenesis consist of extracellular matrix synthesis and turnover, cell adhesion and migration, and epithelial to mesenchymal transition (EMT)) [73]. Among the genes regulated by HIF signaling are phosphoglycerate kinase-1 (PGK), glucose transporter-1 (GLUT1), vascular endothelial growth factor (VEGF), erythropoietin (EPO), and tissue inhibitor of metalloproteinase-1 (TIMP-1)) [73].

Cell death is shown to take part in increasing renal cell depletion in DKD. In vitro studies have shown that hyperglycemia and the high level of glucose death can induce apoptosis. Hyperglycemia occurring in people with diabetes mellitus provokes accelerated apoptosis, leading to cell shrinkage, chromatin condensation, and DNA fragmentation in a variety of cell types, including renal proximal tubular epithelial cells.

Some studies have found that a high production of glucose by kidney cells is able to upregulate proapoptotic molecules (i.e., Bax) and reduce the expression of antiapoptotic molecules (Bcl2 and BclxL). Regulation of apoptotic-related genes by high glucose is very complex. In proximal tubular cells it was demonstrated that high glucose and hyperglycemia cause cell apoptosis through regulation of the Bcl2/caspase 3/PARP pathway [78]. Activated caspase-3 is primarily responsible for the induction of PARP cleavage during cell death [79].

Other players shown to contribute to cell death in DKD are: hypoxia, oxidative stress, inflammatory cytokines, the renin–angiotensin–aldosterone system, advanced glycosylation end products, and glucose degradation products [80,81,82,83].

## 4. Bilirubin Protection against Diabetic Kidney Disease

**Figure 2:**
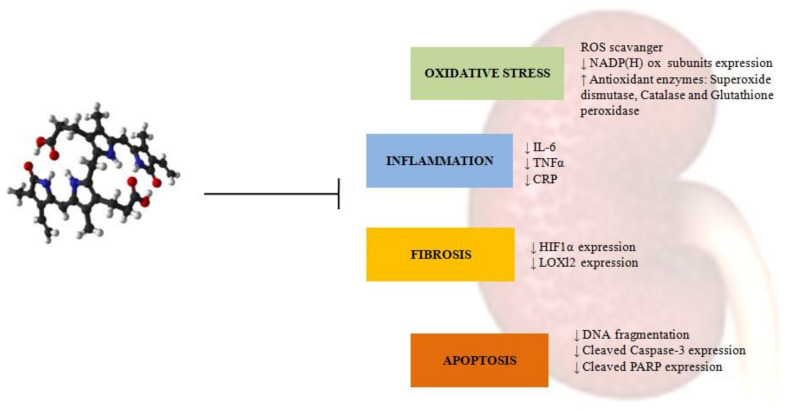
Bilirubin protective effect on mechanisms leading to in diabetic kidney disease. ROS: reactive oxygen species; NADPH: nicotinamide adenine dinucleotide phosphate reduced form; IL-6: interleukin-6; TNFα: tumor necrosis factor alpha; CRP: C-reactive protein; HIF1α: hypoxia inducible factor 1, alpha subunit; LOXl2: lysyl oxidase like 2; DNA: deoxyribonucleic acid; PARP: nuclear poly ADP ribose polymerase.

Bilirubin exerts a preventive role against different metabolic processes known to mediate the onset and the progression of many diseases, particularly vascular diseases, diabetes, metabolic syndrome, and obesity. Mild hyperbilirubinemic Gilbert subjects have a significantly lower risk to be affected by diseases characterized by increased oxidative stress, inflammation, or cell proliferation [8]. Additionally, homozygous Gunn rats combine hyperbilirubinemia with marked anti-inflammatory, antiproliferative [24], antihypertensive [25], blood lipid-modulating properties [84], and with fewer signs of cellular senescence [11]. Even if most of the reported studies referred to experimental animal models (that could be not representative of the human disease), negative correlation of serum bilirubin levels with DKD progression were reported by different clinicalpapers [40,41,42,43]. In chronic hyperglycemia conditions, different pathways are activated and tissue damage occurs, mainly through the induction of oxidative stress, inflammation, fibrosis, and upregulation of some growth factors and their receptors [85]. However, bilirubin can exert its positive and protective effects on these activated and modified pathways in DKD (Figure 2).

### 4.1. Oxidative Stress

Oxidative stress in DKD is a critical factor that pairs hyperglycemia with vascular complications via two major pathways. The first is the metabolic modifications of target molecules present in the tissue, while the second is the alterations in the renal hemodynamics. Excess superoxide leads to DNA damage and activation of nuclear poly ADP ribose polymerase (PARP), which blocks glyceraldehyde-3-phosphate dehydrogenase (GAPDH) activity converting early glycolytic intermediates into pathogenic mediators [86].

Bilirubin is a strong antioxidant, preventing the effects of free radical production and, therefore, oxidative damage [19,31]. Kumar et al. demonstrated that serum bilirubin concentration is negatively correlated with malondialdehyde (MDA), a marker of oxidative stress, while it is positively correlated with superoxide dismutase, catalase, and glutathione peroxidase activity, all antioxidative enzymes [87,88]. Vitek et al. [33] also showed significantly elevated total antioxidant status in GS compared to healthy controls and patients with ischemic heart disease and controls.

In addition, in animal models, antioxidants are effective in treating DKD [89]. Endogenously elevated bilirubin of homozygous Gunn rats specifically protects the vascular compartment from systemic oxidative stress [90]. Bilirubin diminishes kidney damage in cyclosporine-induced nephropathy [91] and ischemia-reperfusion injury [92]. In the Gunn rat diabetic model, bilirubin reduces the streptozotocin-induced pancreatic damage [93]. Inhibition of oxidative stress and the downregulation of NADPH oxidase are the mechanisms demonstrated to protect rodents against DKD [94]. Moderate unconjugated hyperbilirubinemia regressed the vasoconstrictor effect of Ang-II, the decline of glomerular filtration rate, and the increase of renal blood flow and systolic blood pressure by scavenging ROS produced by angiotensin II (Ang-II) [95]. Moreover, UCB affected the intracellular Ca^2+^ imbalance and reversed the expression of vasoconstrictive pre-pro-endothelin induced by Ang-II [96]. Mild hyperbilirubinemia was also shown to lower (Ang II)–dependent hypertension in mice, inhibiting superoxide production [97].

### 4.2. Inflammation

Another potential factor of DKD development is weak chronic inflammation [98]. Experimental findings to date have shown the anti-inflammatory properties of bilirubin in Type-2 diabetes. Bilirubin can affect the expression levels of cell adhesion molecules and complement activity and suppress differentiation of T cells [99,100], the release of interleukin (IL)-2, IL-6, IL-10 and TNF-α and also reduce the expression of major histocompatibility complex class-II expression in macrophages [101]. Not only can bilirubin modulate the immune system, but also the BLVRA enzyme [1]. Hyperbilirubinemic Gunn rats show a reduced inflammatory response in LPS-mediated systemic inflammation [102].

An inverse association of bilirubin with C-reactive protein (CRP), was also reported in various diseases as obesity and diabetes [63,91,103]. A longitudinal cohort study on Korean males examined the correlation between serum bilirubin level and the prevalence of diabetes mellitus and chronic kidney disease in patients with diabetes mellitus in a Korean population. Age, sex, weight, height, waist circumference, history of hypertension and diabetes mellitus, and smoking and alcohol consumption were the clinical parameters examined. Results show that serum bilirubin was inversely associated with levels of insulin resistance and inflammatory markers, serum insulin, and CRP.

However, the anti-inflammatory and immunomodulatory effects of bilirubin are also responsible for the anti-atherosclerotic effects seen in patients with Gilbert’s syndrome [104]. Recently Keum et al. [4] demonstrated that bilirubin nanoparticles (BRNPs), comprising bilirubin, provoke a significant anti-inflammatory activity against various oxidative stress-associated diseases, including hepatic ischemia-reperfusion injury, inflammatory bowel disease, experimental autoimmune encephalomyelitis, allergic lung inflammation, psoriasis, and is let xenotransplantation, counteracting inflammation by scavenging excess ROS.

### 4.3. Fibrosis

Kidney fibrosis is the end-stage pathological change in DKD, and it was shown in animal models that it could be ameliorated by bilirubin treatment. Elevated bilirubin levels are associated with a better renal prognosis and less presence of fibrosis. Furthermore, bilirubin treatment diminishes fibronectin expression in tubular epithelial cells in a dose-dependent manner [105].

HIF-1α can promote renal fibrosis in some kidney diseases. Peritubular capillary loss due to glomerular injury diminishes the oxygen supply leading to chronic interstitial and tubular cell hypoxia in chronic kidney diseases. HIF-1α is expressed across the entire kidney and plays a central role in this hypoxic response of tubular epithelial cells [75]. Prolongated activation of HIF-1α signaling in renal epithelial cells increases maladaptive responses, inducing fibrosis and tissue destruction. HIF-1α functions downstream of a pro-fibrotic signaling cascade initiated by angiotensin II in renal interstitial fibroblasts were recently associated with a consequent activation of the epithelial-mesenchymal transition and excessive accumulation of collagen. Ang II induces dose-dependent HIF-1α and LOXl2 protein expression, acting as a pro-fibrotic marker [106].

Bilirubin seems to modulate the expression of the pro-fibrotic marker. It reduces the induction of pro-fibrotic factor HIF-1α by AGEs and angiotensin II on proximal tubular epithelial cells, and this decrease correlates with downregulation of HIF-1 transcriptional targets, LOXL2 and α-SMA [46]. Furthermore, under physiological oxygen concentration, bilirubin increasesHIF-1α mRNA transcription by reducing ROS and subunits of NADPH oxidase in proximal tubular epithelial cells. Bilirubin also impacts the post-transcriptional modification of HIF-1α protein attenuating ROS effects on the suppression of the P70S6K pathway [103].

### 4.4. Apoptosis

Podocyte apoptosis may exert a crucial role in both early and late stages of DKD, contributing to further reducing the number of the podocytes and the glomerular filtration capacity [66]. Cleavage of PARP by caspase-3 is supposed to be a hallmark of apoptosis [79].

Hyperbilirubinemia reduces the angiotensin II-induced podocytes damage by showing lower DNA fragmentation, cleaved caspase-3, and cleaved PARP induction in hyperbilirubinemic Gunn rats [46]. Also, the bilirubin anti-apoptotic effect was described in the rat model with cyclosporine-induced nephropathy (CsA) in which apoptotic cells in rat kidneys treated with bilirubin were significantly fewer than in controls [107]. Oh et al. [91] reported that bilirubin-treated CsA-induced rats presented a block of apoptosis via the upregulation of antiapoptotic protein bcl-2 and downregulation of proapoptotic Bax expression.

Recent studies highlight the renoprotective function of autophagy in podocytes in models of DKD. Li et al. [108] showed that puerarin, an active compound of radix puerariae, attenuates DKD by promoting autophagy in podocytes. Even resveratrol, another antioxidant compound similar to bilirubin, successfully attenuates apoptosis-activating autophagy in db/db mice and podocytes [109]. In addition, bilirubin was described to beable to induce autophagy as a pro-survival cell mechanism [110] and protected podocytes in models of DKD [46]. Furthermore, heme oxygenase-1 (HMOX-1), an antioxidant enzyme that is induced in response to oxidative stress, promotes autophagy and inhibits apoptosis through the activation of AMPK [111].

## 5. Conclusions and Future Prospective

In the present review, we focused on the beneficial role of mild hyperbilirubinemia against DKD. Recent basic and clinical studies have demonstrated its effects in the onset and progression of renal diseases, pointing out that only little or very mild elevations of serum bilirubin concentrations provide real clinical benefits. Endogenous bilirubin appeared crucial, both as a potential marker for progression and as a therapeutic target for the prevention of DKD. Patients with a low–normal total bilirubin concentration may be managed more aggressively to delay the progression to kidney failure. Further studies are required to determine whether the total bilirubin concentration is a potential therapeutic target for the prevention of CKD. Scientists are taking into consideration modulating plasma bilirubin concentrations to prevent several oxidative stress and inflammation-mediated diseases, such as DKD.

## Data Availability

Not applicable.

## Abbreviations

ABCC2	ATP-binding cassette transporters subfamily C2
AGEs	Advanced glycation end products
AhR	Aryl-hydrocarbon receptor
AMPK	Serine/threonine kinase AMP-activated protein kinase
AngII	Angiotensin II
ApoA1	Apolipoprotein A1
ApoB	Apolipoprotein B
ApoD	Apolipoprotein D
Bax	Bcl-2 Associated X-protein
Bcl2	B-cell lymphoma 2
Bcl-XlB	cell lymphoma-extra large
Bf	Free bilirubin
BLVRA	Biliverdin reductase A enzyme
BRNPs	Bilirubin nanoparticles
CAR	Constitutive androstane receptor
CB	Conjugated bilirubin
CDV	Cardiovascular disease
CKD	Chronic kidney diseases
CNS	Crigler-Najjar syndromes
CO	Carbon monoxide
CRP	C-reactive protein
CsA	Cyclosporine
CXCLs	Soluble C-X-C chemokine ligand
CYP-450	Cytochrome 450
DAG	Diacylglycerol
DKD	Diabetic kidney disease
DNA	Deoxyribonucleic acid
DN	Diabetic nephropathy
EMT	Epithelial to mesenchymal transition
EPO	Erythropoietin
ERK	Extracellular signal-regulated kinase
FABP 1	Fatty acid-binding protein
Fe^2+^	Ferrous ions
GAPDH	Glyceraldehyde-3-phosphate dehydrogenase
GLUT1	Glucose transporter-1
GS	Gilbert syndrome
HDL	High-density lipoprotein
HIF-1α	Hypoxia-inducible factor 1, alpha subunit
HMOX	Hemeoxygenase enzyme
IL-10	Interleukin-10
IL-2	Interleukin-2
IL-6	Interleukin-6
iNOS	Inducible nitric oxide synthase
LDL	Low density lipoproteins
LOXl2	Lysyl Oxidase Like 2
LPS	Lipopolysaccharid
MAPK	Mitogen-activated protein kinase
MDA	Malondialdehyde
MRGPRX4	Mas-related G protein-coupled receptor X4
MRP2	Multidrug resistance-associated protein 2
NADP	Nicotinamide adenine dinucleotide phosphate
NADP+	Nicotinamide adenine dinucleotide phosphate oxidized form
NADPH	Nicotinamide adenine dinucleotide phosphate reduced form
NADPH oxidase	Nicotinamide adenine dinucleotide phosphate oxidase
NF-kβ	Nuclear factor kappa-light-chain-enhancer of activated B cells
NO	Nitric oxide
NOS	Nitric oxide synthase
O_2_	Oxygen
OATP1B1	Organic anion transporting polypeptide 1B1
OMIM	Online Mendelian Inheritance in Man
P70S6K	p70S6 kinase
PARP	Nuclear poly ADP ribose polymerase
PGK	Phosphoglycerate kinase-1
PKC	Protein kinase C
PPARs	Peroxisome proliferator-activated receptors
PXR	Pregnane X receptor
RAAS	Renin–angiotensin–aldosterone system
Raf	Rapidly accelerated fibrosarcoma
ROS	Reactive oxygen species
TGF-β	Transforming growth factor-β
TIF	Tubulointerstitial fibrosis
TIMP-1	Tissue inhibitor of metalloproteinase-1
TNF-α	Tumor necrosis factor alpha
UCB	Unconjugated bilirubin
UDP	Uridine diphosphate;
UDPGA	uridine 5′-diphosphoglucuronic acid
UGT1A1	uridine diphosphate-glucuronosyl transferase 1A1
VEGF	Vascular endothelial growth factor
VLDL	Very low-density lipoprotein
α-SMA	α-smooth muscle actin

## References

[1] Hinds T.D., Stec D.E. (2019). Bilirubin Safeguards Cardiorenal and Metabolic Diseases: A Protective Role in Health. Curr. Hypertens. Rep..

[2] Vitek L., Hubacek J.A., Pajak A., Doryńska A., Kozela M., Eremiasova L., Danzig V., Stefler D., Bobak M. (2019). Association between Plasma Bilirubin and Mortality. Ann. Hepatol..

[3] Alicic R.Z., Rooney M.T., Tuttle K.R. (2017). Diabetic Kidney Disease. Clin. J. Am. Soc. Nephrol. CJASN.

[4] Keum H., Kim D., Kim J., Kim T.W., Whang C.-H., Jung W., Jon S. (2021). A Bilirubin-Derived Nanomedicine Attenuates the Pathological Cascade of Pulmonary Fibrosis. Biomaterials.

[5] Vítek L. (2012). The Role of Bilirubin in Diabetes, Metabolic Syndrome, and Cardiovascular Diseases. Front. Pharmacol..

[6] Vítek L. (2020). Bilirubin as a Signaling Molecule. Med. Res. Rev..

[7] Fevery J. (2008). Bilirubin in Clinical Practice: A Review. Liver Int. Off. J. Int. Assoc. Study Liver.

[8] Vitek L., Bellarosa C., Tiribelli C. (2019). Induction of Mild Hyperbilirubinemia: Hype or Real Therapeutic Opportunity?. Clin. Pharmacol. Ther..

[9] Hahm J.S., Ostrow J.D., Mukerjee P., Celic L. (1992). Ionization and Self-Association of Unconjugated Bilirubin, Determined by Rapid Solvent Partition from Chloroform, with Further Studies of Bilirubin Solubility. J. Lipid Res..

[10] Ahlfors C.E., Wennberg R.P., Ostrow J.D., Tiribelli C. (2009). Unbound (Free) Bilirubin: Improving the Paradigm for Evaluating Neonatal Jaundice. Clin. Chem..

[11] Zelenka J., Dvořák A., Alán L., Zadinová M., Haluzík M., Vítek L. (2016). Hyperbilirubinemia Protects against Aging-Associated Inflammation and Metabolic Deterioration. Oxid. Med. Cell. Longev..

[12] Sundararaghavan V.L., Sindhwani P., Hinds T.D. (2016). Glucuronidation and UGT Isozymes in Bladder: New Targets for the Treatment of Uroepithelial Carcinomas?. Oncotarget.

[13] Méndez-Sánchez N., Qi X., Vitek L., Arrese M. (2019). Evaluating an Outpatient with an Elevated Bilirubin. Am. J. Gastroenterol..

[14] Tiribelli C., Ostrow J.D. (2005). Intestinal Flora and Bilirubin. J. Hepatol..

[15] Sticova E., Jirsa M. (2013). New Insights in Bilirubin Metabolism and Their Clinical Implications. World J. Gastroenterol..

[16] Vítek L., Ostrow J.D. (2009). Bilirubin Chemistry and Metabolism; Harmful and Protective Aspects. Curr. Pharm. Des..

[17] Reddy D.K., Pandey S. (2021). Kernicterus. StatPearls.

[18] Watchko J.F. (2006). Kernicterus and the Molecular Mechanisms of Bilirubin-Induced CNS Injury in Newborns. Neuromolecular Med..

[19] Boon A.-C., Bulmer A.C., Coombes J.S., Fassett R.G. (2014). Circulating Bilirubin and Defense against Kidney Disease and Cardiovascular Mortality: Mechanisms Contributing to Protection in Clinical Investigations. Am. J. Physiol. Renal Physiol..

[20] Gunn C.K. (1944). Hereditary Acholuric Jaundice in the Rat. Can. Med. Assoc. J..

[21] Schutta H.S., Johnson L. (1967). Bilirubin Encephalopathy in the Gunn Rat: A Fine Structure Study of the Cerebellar Cortex. J. Neuropathol. Exp. Neurol..

[22] Gazzin S., Zelenka J., Zdrahalova L., Konickova R., Zabetta C.C., Giraudi P.J., Berengeno A.L., Raseni A., Robert M.C., Vitek L. (2012). Bilirubin Accumulation and Cyp MRNA Expression in Selected Brain Regions of Jaundiced Gunn Rat Pups. Pediatr. Res..

[23] Lanone S., Bloc S., Foresti R., Almolki A., Taillé C., Callebert J., Conti M., Goven D., Aubier M., Dureuil B. (2005). Bilirubin Decreases Nos2 Expression via Inhibition of NAD(P)H Oxidase: Implications for Protection against Endotoxic Shock in Rats. FASEB J. Off. Publ. Fed. Am. Soc. Exp. Biol..

[24] Ollinger R., Bilban M., Erat A., Froio A., McDaid J., Tyagi S., Csizmadia E., Graça-Souza A.V., Liloia A., Soares M.P. (2005). Bilirubin: A Natural Inhibitor of Vascular Smooth Muscle Cell Proliferation. Circulation.

[25] Pflueger A., Croatt A.J., Peterson T.E., Smith L.A., d’Uscio L.V., Katusic Z.S., Nath K.A. (2005). The Hyperbilirubinemic Gunn Rat Is Resistant to the Pressor Effects of Angiotensin II. Am. J. Physiol. Renal Physiol..

[26] Zelenka J., Muchova L., Zelenkova M., Vanova K., Vreman H.J., Wong R.J., Vitek L. (2012). Intracellular Accumulation of Bilirubin as a Defense Mechanism against Increased Oxidative Stress. Biochimie.

[27] Kang S.J., Lee C., Kruzliak P. (2014). Effects of Serum Bilirubin on Atherosclerotic Processes. Ann. Med..

[28] Wagner K.-H., Wallner M., Mölzer C., Gazzin S., Bulmer A.C., Tiribelli C., Vitek L. (2015). Looking to the Horizon: The Role of Bilirubin in the Development and Prevention of Age-Related Chronic Diseases. Clin. Sci. Lond. Engl. 1979.

[29] Wagner K.-H., Shiels R.G., Lang C.A., Seyed Khoei N., Bulmer A.C. (2018). Diagnostic Criteria and Contributors to Gilbert’s Syndrome. Crit. Rev. Clin. Lab. Sci..

[30] Erlinger S., Arias I.M., Dhumeaux D. (2014). Inherited Disorders of Bilirubin Transport and Conjugation: New Insights into Molecular Mechanisms and Consequences. Gastroenterology.

[31] Boon A.-C., Hawkins C.L., Bisht K., Coombes J.S., Bakrania B., Wagner K.-H., Bulmer A.C. (2012). Reduced Circulating Oxidized LDL Is Associated with Hypocholesterolemia and Enhanced Thiol Status in Gilbert Syndrome. Free Radic. Biol. Med..

[32] Vianello E., Zampieri S., Marcuzzo T., Tordini F., Bottin C., Dardis A., Zanconati F., Tiribelli C., Gazzin S. (2018). Histone Acetylation as a New Mechanism for Bilirubin-Induced Encephalopathy in the Gunn Rat. Sci. Rep..

[33] Vítek L., Jirsa M., Brodanová M., Kalab M., Marecek Z., Danzig V., Novotný L., Kotal P. (2002). Gilbert Syndrome and Ischemic Heart Disease: A Protective Effect of Elevated Bilirubin Levels. Atherosclerosis.

[34] Bulmer A.C., Blanchfield J.T., Toth I., Fassett R.G., Coombes J.S. (2008). Improved Resistance to Serum Oxidation in Gilbert’s Syndrome: A Mechanism for Cardiovascular Protection. Atherosclerosis.

[35] Creeden J.F., Gordon D.M., Stec D.E., Hinds T.D. (2021). Bilirubin as a Metabolic Hormone: The Physiological Relevance of Low Levels. Am. J. Physiol. Endocrinol. Metab..

[36] Lee S.J., Jee Y.H., Jung K.J., Hong S., Shin E.S., Jee S.H. (2017). Bilirubin and Stroke Risk Using a Mendelian Randomization Design. Stroke.

[37] Stender S., Frikke-Schmidt R., Nordestgaard B.G., Grande P., Tybjaerg-Hansen A. (2013). Genetically Elevated Bilirubin and Risk of Ischaemic Heart Disease: Three Mendelian Randomization Studies and a Meta-Analysis. J. Intern. Med..

[38] Hinds T.D., Stec D.E. (2018). Bilirubin, a Cardiometabolic Signaling Molecule. Hypertens. Dallas Tex 1979.

[39] Stec D.E., John K., Trabbic C.J., Luniwal A., Hankins M.W., Baum J., Hinds T.D. (2016). Bilirubin Binding to PPARα Inhibits Lipid Accumulation. PLoS ONE.

[40] Chan W.K., Tsai S.-S., Li Y.-R., Chou W.-Y., Chen H.-L., Chen S.-T. (2021). Association between Serum Bilirubin Levels and Progression of Albuminuria in Taiwanese with Type 2 Diabetes Mellitus. Biomed. J..

[41] Liu M., Li J., Lv X., He Y. (2018). Bilirubin and Its Changes Were Negatively Associated with Diabetic Kidney Disease Incidence and Progression: A Five-Year’s Cohort Study Based on 5323 Chinese Male Diabetic Patients. J. Diabetes Complicat..

[42] Ahn K.H., Kim S.S., Kim W.J., Kim J.H., Nam Y.J., Park S.B., Jeon Y.K., Kim B.H., Kim I.J., Kim Y.K. (2017). Low Serum Bilirubin Level Predicts the Development of Chronic Kidney Disease in Patients with Type 2 Diabetes Mellitus. Korean J. Intern. Med..

[43] Uludag K., Oguzhan N., Arıkan T., Boz G. (2018). Serum Bilirubin Level and Its Impact on the Progression of Chronic Kidney Disease. Int. Urol. Nephrol..

[44] Li X., Zhang L., Chen H., Guo K., Yu H., Zhou J., Li M., Li Q., Li L., Yin J. (2017). Relationship between Serum Bilirubin Concentrations and Diabetic Nephropathy in Shanghai Han’s Patients with Type 1 Diabetes Mellitus. BMC Nephrol..

[45] Rigato I., Ostrow J.D., Tiribelli C. (2005). Bilirubin and the Risk of Common Non-Hepatic Diseases. Trends Mol. Med..

[46] Bianco A., Pinci S., Tiribelli C., Bellarosa C. (2021). Life-Long Hyperbilirubinemia Exposure and Bilirubin Priming Prevent In Vitro Metabolic Damage. Front. Pharmacol..

[47] Brito M.A., Lima S., Fernandes A., Falcão A.S., Silva R.F.M., Butterfield D.A., Brites D. (2008). Bilirubin Injury to Neurons: Contribution of Oxidative Stress and Rescue by Glycoursodeoxycholic Acid. Neurotoxicology.

[48] Kumar S., Guha M., Choubey V., Maity P., Srivastava K., Puri S.K., Bandyopadhyay U. (2008). Bilirubin Inhibits Plasmodium Falciparum Growth through the Generation of Reactive Oxygen Species. Free Radic. Biol. Med..

[49] Oakes G.H., Bend J.R. (2005). Early Steps in Bilirubin-Mediated Apoptosis in Murine Hepatoma (Hepa 1c1c7) Cells Are Characterized by Aryl Hydrocarbon Receptor-Independent Oxidative Stress and Activation of the Mitochondrial Pathway. J. Biochem. Mol. Toxicol..

[50] Bianco A., Dvořák A., Capková N., Gironde C., Tiribelli C., Furger C., Vitek L., Bellarosa C. (2020). The Extent of Intracellular Accumulation of Bilirubin Determines Its Anti- or Pro-Oxidant Effect. Int. J. Mol. Sci..

[51] Jangi S., Otterbein L., Robson S. (2013). The Molecular Basis for the Immunomodulatory Activities of Unconjugated Bilirubin. Int. J. Biochem. Cell Biol..

[52] Barateiro A., Vaz A.R., Silva S.L., Fernandes A., Brites D. (2012). ER Stress, Mitochondrial Dysfunction and Calpain/JNK Activation Are Involved in Oligodendrocyte Precursor Cell Death by Unconjugated Bilirubin. Neuromolecular Med..

[53] Zhu H., Wang J., Jiang H., Ma Y., Pan S., Reddy S., Sun X. (2010). Bilirubin Protects Grafts against Nonspecific Inflammation-Induced Injury in Syngeneic Intraportal Islet Transplantation. Exp. Mol. Med..

[54] Tran D.T., Jeong Y.Y., Kim J.M., Bae H.B., Son S.K., Kwak S.H. (2020). The Anti-Inflammatory Role of Bilirubin on “Two-Hit” Sepsis Animal Model. Int. J. Mol. Sci..

[55] Li Y., Huang B., Ye T., Wang Y., Xia D., Qian J. (2020). Physiological Concentrations of Bilirubin Control Inflammatory Response by Inhibiting NF-ΚB and Inflammasome Activation. Int. Immunopharmacol..

[56] Mazzone G.L., Rigato I., Ostrow J.D., Tiribelli C. (2009). Bilirubin Effect on Endothelial Adhesion Molecules Expression Is Mediated by the NF-KappaB Signaling Pathway. Biosci. Trends.

[57] Gundamaraju R., Vemuri R., Chong W.C., Bulmer A.C., Eri R. (2019). Bilirubin Attenuates ER Stress-Mediated Inflammation, Escalates Apoptosis and Reduces Proliferation in the LS174T Colonic Epithelial Cell Line. Int. J. Med. Sci..

[58] Hinds T.D., Gordon D.M., Combs S.D., Stec D.E. (2018). Bilirubin, a Novel Endocrine Hormone with Fat Burning Properties. FASEB J..

[59] Vítek L., Tiribelli C. (2021). Bilirubin: The Yellow Hormone?. J. Hepatol..

[60] Žiberna L., Jenko-Pražnikar Z., Petelin A. (2021). Serum Bilirubin Levels in Overweight and Obese Individuals: The Importance of Anti-Inflammatory and Antioxidant Responses. Antioxid. Basel Switz..

[61] Persson F., Rossing P. (2018). Diagnosis of Diabetic Kidney Disease: State of the Art and Future Perspective. Kidney Int. Suppl..

[62] Slyne J., Slattery C., McMorrow T., Ryan M.P. (2015). New Developments Concerning the Proximal Tubule in Diabetic Nephropathy: In Vitro Models and Mechanisms. Nephrol. Dial. Transplant..

[63] Amatruda M., Gembillo G., Giuffrida A.E., Santoro D., Conti G. (2021). The Aggressive Diabetic Kidney Disease in Youth-Onset Type 2 Diabetes: Pathogenetic Mechanisms and Potential Therapies. Medicina.

[64] Giandalia A., Giuffrida A.E., Gembillo G., Cucinotta D., Squadrito G., Santoro D., Russo G.T. (2021). Gender Differences in Diabetic Kidney Disease: Focus on Hormonal, Genetic and Clinical Factors. Int. J. Mol. Sci..

[65] Santoro D., Torreggiani M., Pellicanò V., Cernaro V., Messina R.M., Longhitano E., Siligato R., Gembillo G., Esposito C., Piccoli G.B. (2021). Kidney Biopsy in Type 2 Diabetic Patients: Critical Reflections on Present Indications and Diagnostic Alternatives. Int. J. Mol. Sci..

[66] Bose M., Almas S., Prabhakar S. (2017). Wnt Signaling and Podocyte Dysfunction in Diabetic Nephropathy. J. Investig. Med. Off. Publ. Am. Fed. Clin. Res..

[67] Maezawa Y., Takemoto M., Yokote K. (2015). Cell Biology of Diabetic Nephropathy: Roles of Endothelial Cells, Tubulointerstitial Cells and Podocytes. J. Diabetes Investig..

[68] Xiong Y., Zhou L. (2019). The Signaling of Cellular Senescence in Diabetic Nephropathy. Oxid. Med. Cell. Longev..

[69] Duran-Salgado M.B., Rubio-Guerra A.F. (2014). Diabetic Nephropathy and Inflammation. World J. Diabetes.

[70] Talsma D.T., Katta K., Ettema M.A.B., Kel B., Kusche-Gullberg M., Daha M.R., Stegeman C.A., van den Born J., Wang L. (2018). Endothelial Heparan Sulfate Deficiency Reduces Inflammation and Fibrosis in Murine Diabetic Nephropathy. Lab. Invest..

[71] Taslipinar A., Yaman H., Yilmaz M.I., Demirbas S., Saglam M., Taslipinar M.Y., Agilli M., Kurt Y.G., Sonmez A., Azal O. (2011). The Relationship between Inflammation, Endothelial Dysfunction and Proteinuria in Patients with Diabetic Nephropathy. Scand. J. Clin. Lab. Invest..

[72] Feigerlová E., Battaglia-Hsu S.-F. (2017). IL-6 Signaling in Diabetic Nephropathy: From Pathophysiology to Therapeutic Perspectives. Cytokine Growth Factor Rev..

[73] Haase V.H. (2012). Hypoxia-Inducible Factor Signaling in the Development of Kidney Fibrosis. Fibrogenesis Tissue Repair.

[74] Higgins D.F., Kimura K., Bernhardt W.M., Shrimanker N., Akai Y., Hohenstein B., Saito Y., Johnson R.S., Kretzler M., Cohen C.D. (2007). Hypoxia Promotes Fibrogenesis in Vivo via HIF-1 Stimulation of Epithelial-to-Mesenchymal Transition. J. Clin. Investig..

[75] Kimura K., Iwano M., Higgins D.F., Yamaguchi Y., Nakatani K., Harada K., Kubo A., Akai Y., Rankin E.B., Neilson E.G. (2008). Stable Expression of HIF-1alpha in Tubular Epithelial Cells Promotes Interstitial Fibrosis. Am. J. Physiol. Renal Physiol..

[76] Huang H., Fan Y., Gao Z., Wang W., Shao N., Zhang L., Yang Y., Zhu W., Chen Z., Hu J. (2019). HIF-1α Contributes to Ang II-Induced Inflammatory Cytokine Production in Podocytes. BMC Pharmacol. Toxicol..

[77] Schietke R., Warnecke C., Wacker I., Schödel J., Mole D.R., Campean V., Amann K., Goppelt-Struebe M., Behrens J., Eckardt K.-U. (2010). The Lysyl Oxidases LOX and LOXL2 Are Necessary and Sufficient to Repress E-Cadherin in Hypoxia: Insights into Cellular Transformation Processes Mediated by HIF-1. J. Biol. Chem..

[78] Habib S.L. (2013). Diabetes and Renal Tubular Cell Apoptosis. World J. Diabetes.

[79] Jagtap P., Szabó C. (2005). Poly(ADP-Ribose) Polymerase and the Therapeutic Effects of Its Inhibitors. Nat. Rev. Drug Discov..

[80] Lee S.H., Yoo T.-H., Nam B.-Y., Kim D.K., Li J.J., Jung D.-S., Kwak S.-J., Ryu D.-R., Han S.H., Lee J.E. (2009). Activation of Local Aldosterone System within Podocytes Is Involved in Apoptosis under Diabetic Conditions. Am. J. Physiol. Renal Physiol..

[81] Liu F., Brezniceanu M.-L., Wei C.-C., Chénier I., Sachetelli S., Zhang S.-L., Filep J.G., Ingelfinger J.R., Chan J.S.D. (2008). Overexpression of Angiotensinogen Increases Tubular Apoptosis in Diabetes. J. Am. Soc. Nephrol. JASN.

[82] Sanchez-Niño M.-D., Benito-Martin A., Ortiz A. (2010). New Paradigms in Cell Death in Human Diabetic Nephropathy. Kidney Int..

[83] Cardoso V.G., Gonçalves G.L., Costa-Pessoa J.M., Thieme K., Lins B.B., Casare F.A.M., de Ponte M.C., Camara N.O.S., Oliveira-Souza M. (2018). Angiotensin II-Induced Podocyte Apoptosis Is Mediated by Endoplasmic Reticulum Stress/PKC-δ/P38 MAPK Pathway Activation and Trough Increased Na+/H+ Exchanger Isoform 1 Activity. BMC Nephrol..

[84] Wallner M., Marculescu R., Doberer D., Wolzt M., Wagner O., Vitek L., Bulmer A.C., Wagner K.-H. (2013). Protection from Age-Related Increase in Lipid Biomarkers and Inflammation Contributes to Cardiovascular Protection in Gilbert’s Syndrome. Clin. Sci. Lond. Engl. 1979.

[85] Bahreini E., Rezaei-Chianeh Y., Nabi-Afjadi M. (2021). Molecular Mechanisms Involved in Intrarenal Renin-Angiotensin and Alternative Pathways in Diabetic Nephropathy—A Review. Rev. Diabet. Stud. RDS.

[86] Yuan T., Yang T., Chen H., Fu D., Hu Y., Wang J., Yuan Q., Yu H., Xu W., Xie X. (2018). New Insights into Oxidative Stress and Inflammation during Diabetes Mellitus-Accelerated Atherosclerosis. Redox Biol..

[87] Kumar A., Pant P., Basu S., Rao G.R.K., Khanna H.D. (2007). Oxidative Stress in Neonatal Hyperbilirubinemia. J. Trop. Pediatr..

[88] Yan P., Zhang Z., Miao Y., Xu Y., Zhu J., Wan Q. (2019). Physiological Serum Total Bilirubin Concentrations Were Inversely Associated with Diabetic Peripheral Neuropathy in Chinese Patients with Type 2 Diabetes: A Cross-Sectional Study. Diabetol. Metab. Syndr..

[89] Jha J.C., Banal C., Chow B.S.M., Cooper M.E., Jandeleit-Dahm K. (2016). Diabetes and Kidney Disease: Role of Oxidative Stress. Antioxid. Redox Signal..

[90] Boon A.-C., Lam A.K., Gopalan V., Benzie I.F., Briskey D., Coombes J.S., Fassett R.G., Bulmer A.C. (2015). Endogenously Elevated Bilirubin Modulates Kidney Function and Protects from Circulating Oxidative Stress in a Rat Model of Adenine-Induced Kidney Failure. Sci. Rep..

[91] Oh S.W., Lee E.S., Kim S., Na K.Y., Chae D.W., Kim S., Chin H.J. (2013). Bilirubin Attenuates the Renal Tubular Injury by Inhibition of Oxidative Stress and Apoptosis. BMC Nephrol..

[92] Adin C.A., Croker B.P., Agarwal A. (2005). Protective Effects of Exogenous Bilirubin on Ischemia-Reperfusion Injury in the Isolated, Perfused Rat Kidney. Am. J. Physiol. Renal Physiol..

[93] Fu Y.Y., Kang K.J., Ahn J.M., Kim H.-R., Na K.Y., Chae D.-W., Kim S., Chin H.J. (2010). Hyperbilirubinemia Reduces the Streptozotocin-Induced Pancreatic Damage through Attenuating the Oxidative Stress in the Gunn Rat. Tohoku J. Exp. Med..

[94] Fujii M., Inoguchi T., Sasaki S., Maeda Y., Zheng J., Kobayashi K., Takayanagi R. (2010). Bilirubin and Biliverdin Protect Rodents against Diabetic Nephropathy by Downregulating NAD(P)H Oxidase. Kidney Int..

[95] Vera T., Stec D.E. (2010). Moderate Hyperbilirubinemia Improves Renal Hemodynamics in ANG II-Dependent Hypertension. Am. J. Physiol. Regul. Integr. Comp. Physiol..

[96] Stec D.E., Hosick P.A., Granger J.P. (2012). Bilirubin, Renal Hemodynamics, and Blood Pressure. Front. Pharmacol..

[97] Stec D.E., Storm M.V., Pruett B.E., Gousset M.U. (2013). Antihypertensive Actions of Moderate Hyperbilirubinemia: Role of Superoxide Inhibition. Am. J. Hypertens..

[98] Herder C., Kannenberg J.M., Huth C., Carstensen-Kirberg M., Rathmann W., Koenig W., Heier M., Püttgen S., Thorand B., Peters A. (2017). Proinflammatory Cytokines Predict the Incidence and Progression of Distal Sensorimotor Polyneuropathy: KORA F4/FF4 Study. Diabetes Care.

[99] Kim E.S., Lee S.W., Mo E.Y., Moon S.D., Han J.H. (2015). Inverse Association between Serum Total Bilirubin Levels and Diabetic Peripheral Neuropathy in Patients with Type 2 Diabetes. Endocrine.

[100] Chen J., Wang J., Zhang X., Zhu H. (2017). Inverse Relationship Between Serum Bilirubin Levels and Diabetic Foot in Chinese Patients with Type 2 Diabetes Mellitus. Med. Sci. Monit..

[101] Jin J., Wang W., Gu T., Chen C., Sun J., Chen W., Bi Y., Zhu D. (2019). Low Serum Bilirubin Levels Contribute to the Presence and Progression of Distal Symmetrical Polyneuropathy in Chinese Patients with Type 2 Diabetes. Diabetes Metab..

[102] Valaskova P., Dvorak A., Lenicek M., Zizalova K., Kutinova-Canova N., Zelenka J., Cahova M., Vitek L., Muchova L. (2019). Hyperbilirubinemia in Gunn Rats Is Associated with Decreased Inflammatory Response in LPS-Mediated Systemic Inflammation. Int. J. Mol. Sci..

[103] Kim S.G., Ahn S.-Y., Lee E.S., Kim S., Na K.Y., Chae D.-W., Chin H.J. (2014). Bilirubin Activates Transcription of HIF-1α in Human Proximal Tubular Cells Cultured in the Physiologic Oxygen Content. J. Korean Med. Sci..

[104] Bulmer A.C., Bakrania B., Du Toit E.F., Boon A.-C., Clark P.J., Powell L.W., Wagner K.-H., Headrick J.P. (2018). Bilirubin Acts as a Multipotent Guardian of Cardiovascular Integrity: More than Just a Radical Idea. Am. J. Physiol. Heart Circ. Physiol..

[105] Park S., Kim D.H., Hwang J.H., Kim Y.-C., Kim J.H., Lim C.S., Kim Y.S., Yang S.H., Lee J.P. (2017). Elevated Bilirubin Levels Are Associated with a Better Renal Prognosis and Ameliorate Kidney Fibrosis. PLoS ONE.

[106] Wang Z., Tang L., Zhu Q., Yi F., Zhang F., Li P.-L., Li N. (2011). Hypoxia-Inducible Factor-1α Contributes to the Profibrotic Action of Angiotensin II in Renal Medullary Interstitial Cells. Kidney Int..

[107] Oh S.W., Ahn J.M., Lee Y.-M., Kim S., Chin H.J., Chae D.-W., Na K.Y. (2012). Activation of Hypoxia-Inducible Factor by Cobalt Is Associated with the Attenuation of Tissue Injury and Apoptosis in Cyclosporine-Induced Nephropathy. Tohoku J. Exp. Med..

[108] Li X., Zhu Q., Zheng R., Yan J., Wei M., Fan Y., Deng Y., Zhong Y. (2020). Puerarin Attenuates Diabetic Nephropathy by Promoting Autophagy in Podocytes. Front. Physiol..

[109] Huang S.-S., Ding D.-F., Chen S., Dong C.-L., Ye X.-L., Yuan Y.-G., Feng Y.-M., You N., Xu J.-R., Miao H. (2017). Resveratrol Protects Podocytes against Apoptosis via Stimulation of Autophagy in a Mouse Model of Diabetic Nephropathy. Sci. Rep..

[110] Qaisiya M., Mardešić P., Pastore B., Tiribelli C., Bellarosa C. (2017). The Activation of Autophagy Protects Neurons and Astrocytes against Bilirubin-Induced Cytotoxicity. Neurosci. Lett..

[111] Dong C., Zheng H., Huang S., You N., Xu J., Ye X., Zhu Q., Feng Y., You Q., Miao H. (2015). Heme Oxygenase-1 Enhances Autophagy in Podocytes as a Protective Mechanism against High Glucose-Induced Apoptosis. Exp. Cell Res..

